# Biportal endoscopic spinal surgery for thoracic ossification of the ligamentum flavum: a study of different classification types and surgical outcomes

**DOI:** 10.3389/fneur.2026.1793263

**Published:** 2026-06-05

**Authors:** Honggang Wang, Dongqing He, Ruxing Liu, Jie Yuan, Yongfeng Wang

**Affiliations:** 1 Department of Orthopedics, Second Hospital of Shanxi Medical University, Taiyuan, China; 2 Department of Shanxi key Laboratory of Bone and Soft Tissue Injury Repair, Second Hospital of Shanxi Medical University, Taiyuan, China

**Keywords:** biportal endoscopic spinal surgery, clinical outcome, minimally invasive surgery, ossification of ligamentum flavum, thoracic myelopathy

## Abstract

**Background:**

This study aimed to assess potential differences in the clinical effectiveness of biportal endoscopic spinal surgery (BESS) for treating various axial classifications of ossification of the ligamentum flavum (OLF).

**Methods:**

We retrospectively reviewed patients with thoracic OLF who underwent BESS between January 2023 and June 2024. The OLF type was classified based on preoperative computed tomography (CT) scans, and patients were divided into two groups: non-fused and fused. To evaluate primary radiographic outcomes, we measured changes in the cross-sectional area (CSA) and anteroposterior diameter (APD) on both preoperative and postoperative magnetic resonance imaging (MRI) scans. Clinical outcomes were assessed using the Visual Analog Scale (VAS), the modified Japanese Orthopedic Association (mJOA) score, and the MacNab criteria.

**Results:**

This study included 37 patients: 19 in the non-fused group and 18 in the fused group. Both groups showed significant improvements in VAS and mJOA scores from preoperative baseline values (*p* < 0.05). No significant differences in recovery rate were observed between the two groups at any postoperative time point (*p* > 0.05). The increase in APD was significantly greater in the fused group than in the non-fused group (*p* < 0.05), whereas the increase in CSA was similar between the two groups (*p* > 0.05). The fused group had longer operative times and a higher incidence of complications than the non-fused group.

**Conclusion:**

BESS produces comparable short-term clinical outcomes for fused and non-fused thoracic OLF; however, complex cases require careful patient evaluation and may be best managed by experienced endoscopic surgeons.

## 1 Introduction

Ossification of the ligamentum flavum (OLF) results from progressive endochondral ossification and most commonly occurs in the lower thoracic spine (T10–T12) (1). Thoracic OLF has an insidious onset and is often accompanied by degenerative changes in the cervical and lumbar spine, making early diagnosis challenging. Timely surgical intervention is essential for achieving a favorable neurological recovery. Surgical options include open laminectomy and endoscopic decompression (2).

Open laminectomy for OLF may compromise the stability of the posterior column of the spine and increase the likelihood of postoperative thoracic kyphotic deformity. Additionally, the risks of intraoperative dural tears and cerebrospinal fluid leakage are relatively high (3). In contrast, endoscopic decompression is performed under direct visualization, reducing intraoperative tissue damage and perioperative complications. Both microendoscopic and percutaneous endoscopic decompression are safe and effective treatments for OLF (4–6). Biportal endoscopic spinal surgery (BESS) is not limited by the working channel, providing a wider surgical field and greater operative flexibility. BESS offers certain advantages for treating spinal stenosis and has gained widespread recognition in this field (7, 8).

**Figure 1:**
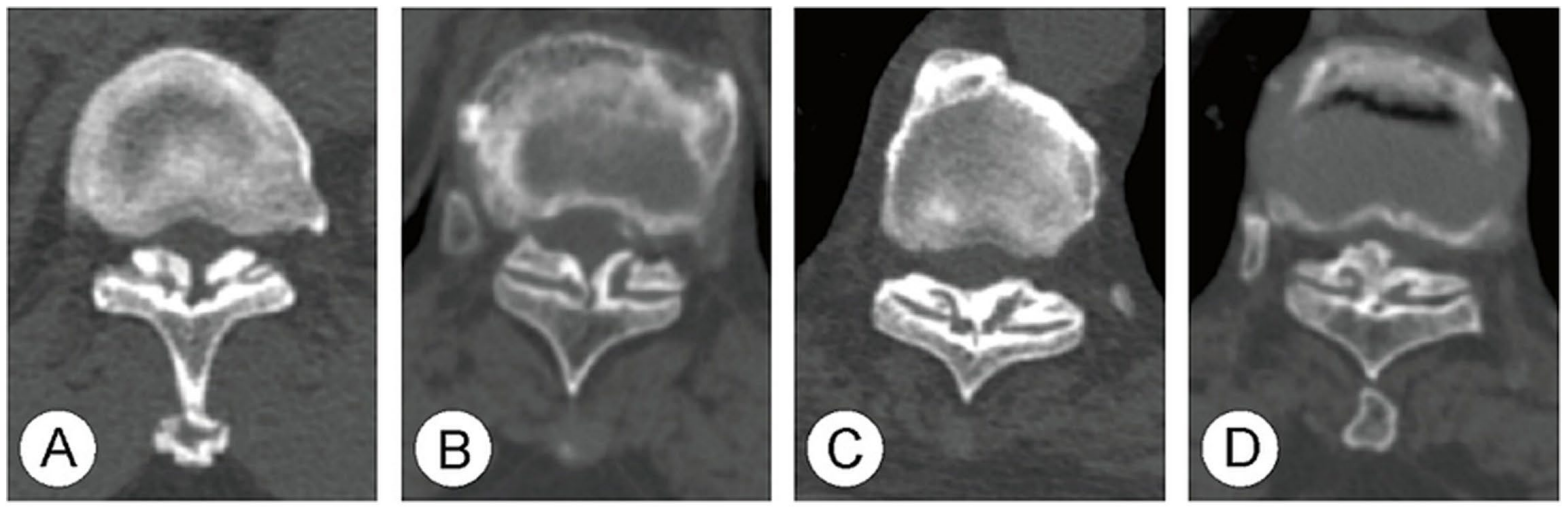
Thoracic OLF was classified into non-fused types **(A,B)** and fused types **(C,D)** based on the results of axial CT scans.

Based on CT scans, Yoon et al. classified the axial configuration of OLF into non-fused and fused types, and two studies showed that after open laminectomy, the recovery rate in non-fused OLF was significantly higher than in fused OLF (Figure 1) (9, 10). However, previous studies have not compared the clinical effectiveness of BESS across different axial types of OLF. This study aimed to compare the clinical effectiveness of BESS between fused and non-fused OLF.

## 2 Materials and methods

All methods were carried out in accordance with the Declaration of Helsinki. This study received approval from the Ethics Committee of the Second Hospital of Shanxi Medical University (2025YX No. 304).

### 2.1 Patient population

We conducted a retrospective study of patients with OLF who underwent BESS at our hospital from January 2023 to June 2024. The inclusion criteria were: (1) symptoms of myelopathy in the back and legs lasting more than six weeks that did not respond to conservative treatment; (2) single-segment OLF confirmed by magnetic resonance imaging (MRI) and computed tomography (CT); and (3) underwent BESS with at least one year of follow-up. The exclusion criteria included: (1) other spinal diseases such as cervical spondylotic myelopathy and lumbar spinal stenosis; (2) the need for decompression surgery across two or more consecutive segments; (3) OLF without clinical symptoms; and (4) surgical contraindications or refusal to undergo surgery. All procedures were performed by a single surgeon with three years of experience in biportal endoscopic techniques.

### 2.2 Surgical procedure

**Figure 2:**
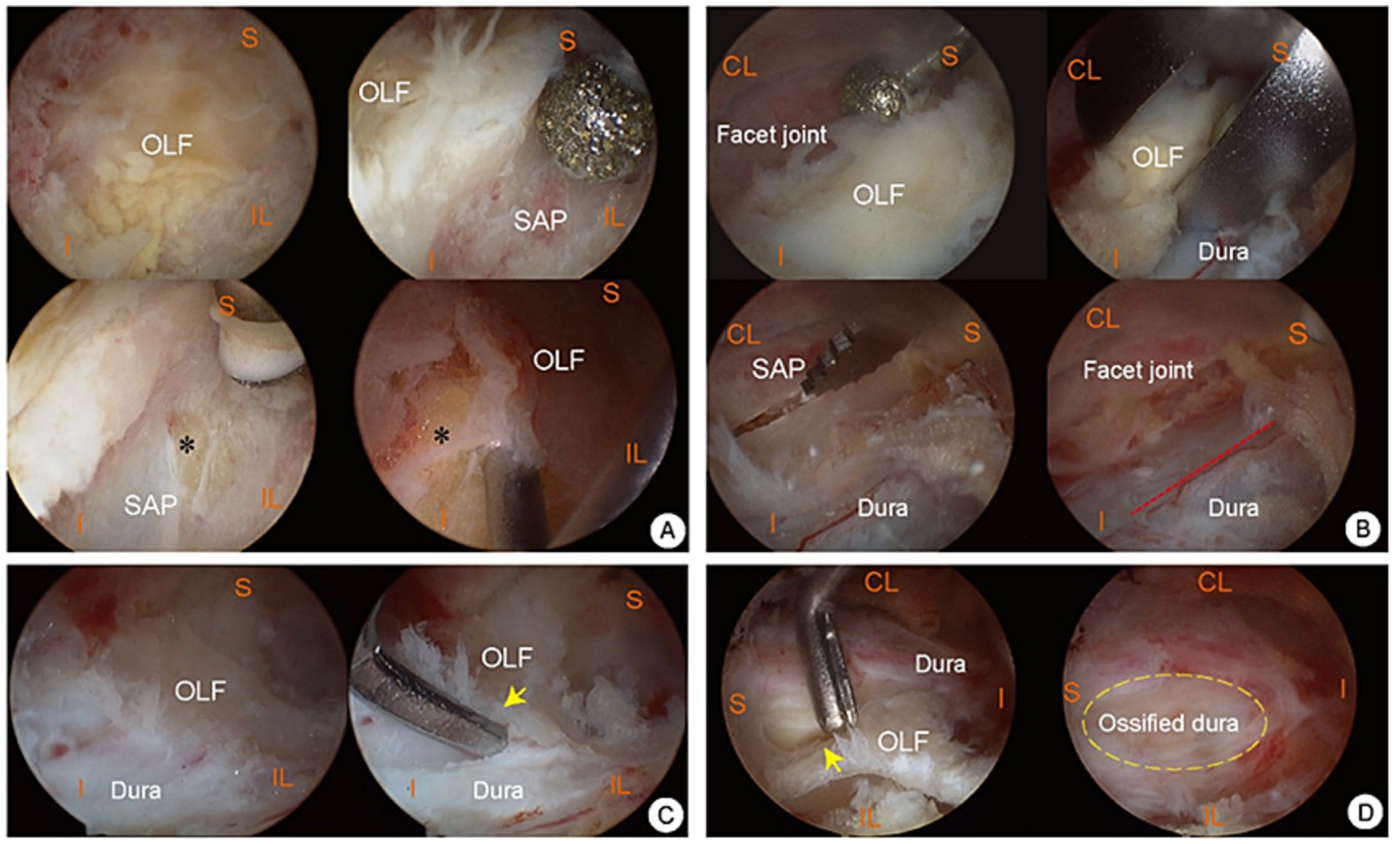
Intraoperative images of the biportal endoscopic spinal surgery procedure **(A–D)**. **(A)** After ipsilateral laminotomy, the ossification of the ligamentum flavum (OLF) was widely exposed (upper left). Ipsilateral superior articular process (SAP) drilling was performed (upper right) until it appeared as a thin paper, and epidural fat was observed (lower left). A blunt hook was used to separate the dura mater from the OLF (lower right). **(B)** The contralateral facet joint was drilled to expose the contralateral SAP (upper left). The ossified flap was removed when it started bobbing (upper right). Contralateral SAP was cut using an ultrasonic osteotome (lower left). At the end of decompression, the contralateral dural sac was sufficiently expanded (lower right). **(C)** OLF is closely adhered to the dura mater (left). Under magnified endoscopic visualization, a blunt hook was used to separate adhesions between the ossified inner layer and the dura mater (yellow arrowhead). **(D)** Separation between the dura and OLF mass is difficult (yellow arrowhead). The ossified tissue was completely separated from the surrounding tissue and retained with the dura mater (right). Yellow dotted line: ossified dura. Red dotted line: midline. Black asterisk: epidural fat. S, superior; I, inferior; CL, contralateral; IL, ipsilateral.

Under general anesthesia, the patient was placed in the prone position, and neurophysiological monitoring was performed. The target level was confirmed using C-arm fluoroscopy, and the skin was marked. The surgical site was thoroughly prepared, and two 1 cm longitudinal incisions were carefully made along the medial borders of the adjacent pedicles at the designated spinal level. Under fluoroscopic guidance, a Kirschner wire was used to locate the lower edge of the upper lamina. A high-speed drill was then used to create a hole along the medial side of the facet joint, advancing from the periphery toward the center until the OLF was thinned to a paper-thin thickness, revealing the epidural fat. A blunt hook was gently used to separate the dura and ossified ligamentum flavum, allowing for OLF removal (Figure 2A). After decompressing the ipsilateral side, a high-speed drill or ultrasonic osteotome was used to remove the base of the spinous process and the ventral part of the contralateral lamina, freeing the contralateral OLF. Finally, adhesions were dissected, and the OLF was removed, ensuring good dural expansion (Figure 2B). The incision was sutured, and a drainage tube was inserted.

The fused OLF typically closely adheres to the dura mater, necessitating careful dissection of the dura from the ossified tissue under magnified endoscopic visualization (Figure 2C). If complete separation between the dura and OLF mass is difficult, excessive dissection may result in a dural tear. In such cases, it is advisable to separate the ossified material from the surrounding tissues and allow it to float above the dura (Figure 2D).

### 2.3 Outcome assessment

**Figure 3:**
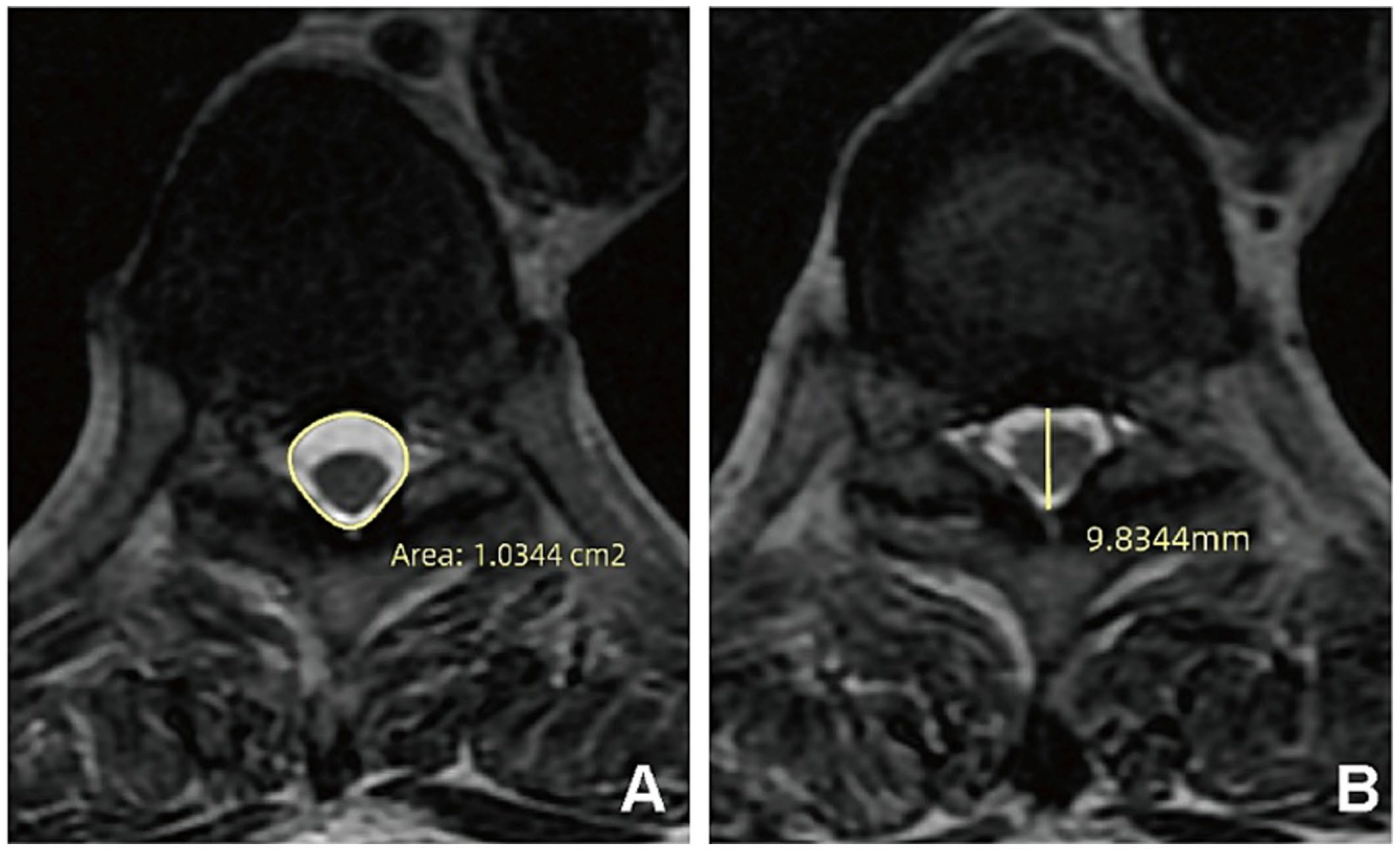
Measurement of cross-sectional area **(A)** and anteroposterior diameter **(B)** on preoperative and postoperative MRI.

We gathered clinical and demographic data, including sex, age, operated segment, surgical method, operative duration, length of hospital stay, and postoperative complications. All patients underwent preoperative X-ray, CT, and MRI scans to identify the type of OLF and determine the appropriate surgical approach. Clinical outcomes were assessed using the Visual Analog Scale (VAS), the modified Japanese Orthopedic Association (mJOA) score, and the MacNab criteria. The recovery rate (RR) was calculated as: RR = (postoperative mJOA - preoperative mJOA) / (11 - preoperative mJOA) × 100%. Blinded to the patients’ clinical information, two independent investigators who were not involved in the surgery measured the cross-sectional area (CSA) and anteroposterior diameter (APD) from preoperative and 3-month postoperative MRI scans (Figure 3) (9, 11). Both observers double-checked each measurement, and the analysis was based on the average of the two measurements.

### 2.4 Statistical analysis

Statistical analysis was performed using SPSS version 27.0 (IBM, Armonk, NY, USA). Continuous data that follow a normal distribution are shown as mean ± standard deviation. A repeated-measures ANCOVA was performed, with the corresponding baseline value as a covariate, to assess the main effects of time and group, as well as their interaction. Mauchly’s test of sphericity was performed, and the Greenhouse–Geisser correction was applied when necessary. A repeated-measures ANOVA was used for RR, which inherently accounts for baseline. Independent sample t-tests compared other continuous variables between the fused and non-fused groups. Categorical variables were analyzed using the *χ^2^* test or Fisher’s exact test when the expected count was less than 5. Interobserver reliability for radiological measurements was assessed with the intraclass correlation coefficient (ICC). A significance level of *α* = 0.05 was set, with *p* < 0.05 indicating statistical significance.

## 3 Results

### 3.1 Baseline characteristics

A total of 37 patients with thoracic OLF who underwent BESS were included in the study: 19 with non-fused OLF and 18 with fused OLF. The non-fused group included 12 males and 7 females, with an average age of 62.6 ± 7.5 years and a mean follow-up period of 15.4 ± 4.0 months. The fused group consisted of 12 males and 6 females, with an average age of 66.3 ± 9.2 years and a mean follow-up of 14.4 ± 3.6 months. No statistically significant differences were observed between the two groups concerning age, sex distribution, follow-up duration, symptom duration, or length of hospital stay. The most commonly affected levels were T10-11 and T11-12 in both groups. Notably, the fused group exhibited a significantly longer operative time than the non-fused group (*p* < 0.001) (Table 1).

**Table 1:** Patient and surgical data.

Variable	Non-fused group (*n* = 19)	Fused group (*n* = 18)	*p* value
Age (years)	62.6 ± 7.5	66.3 ± 9.2	0.192
Sex(male:female)	12:7	12:6	
Follow-up (months)	15.4 ± 4.0	14.4 ± 3.6	0.437
Operating levels			
T2-3	1	1	
T9-10	0	2	
T10-11	6	8	
T11-12	12	7	
Symptom duration (months)	14.2 ± 9.4	11.4 ± 7.5	0.340
Hospital stays (day)	8.4 ± 1.7	8.5 ± 1.5	0.805
Preoperative symptoms			
Sensory deficit	17/19	16/18	
Motor deficit	12/19	13/18	
Sphincter dysfunction	1/19	2/18	
Operation time (min)	61.8 ± 10.8	94.9 ± 11.1	<0.001
Complication			
Insufficient decompression	0	2	
Excessive facet resection	0	2	
Spinous process fracture	0	1	
Dural tear	0	2	
Neurological deterioration	1	0	

Values are presented as numbers or mean ± standard deviation.

### 3.2 Clinical outcomes

Both groups showed a significant decrease in VAS scores during follow-up. After adjusting for preoperative values, the repeated-measures ANCOVA revealed a significant main effect of time (*p* < 0.001, *F* = 19.738); however, no significant main effect of group (*p* = 0.061, *F* = 3.744) or time-by-group interaction (*p* = 0.397, *F* = 0.824) was observed, indicating that both groups displayed similar patterns of pain alleviation over the study period. Similarly, mJOA scores improved significantly after adjusting for baseline. The repeated-measures ANCOVA showed a highly significant main effect of time (*p* < 0.001, *F* = 12.867). The main effect of group was not significant (*p* = 0.112, *F* = 2.664), and the time-by-group interaction was also not significant (*p* = 0.981, *F* = 0.010), indicating that the pattern of improvement over time was similar between the two groups. The RR increased steadily in both groups over the follow-up period, with no significant differences between the groups at any time point. ANOVA revealed a significant main effect of time (*p* < 0.001, *F* = 51.159), but no significant main effect of group (*p* = 0.204, *F* = 1.673) or interaction (*p* = 0.634, *F* = 0.403) (Table 2).

**Table 2:** Clinical outcomes.

Variable	Group	Time point	Unadjusted mean ± SD	Adjusted mean (95% CI)
VAS leg pain	Non-fused group	Preoperative	4.7 ± 2.4	—
		3 days postoperatively	3.1 ± 1.4^†,^	3.0 (2.4–3.6)
		3 months postoperatively	1.3 ± 0.9^†,‡^	1.2 (0.9–1.5)
		1 year postoperatively	0.8 ± 0.7^†,‡,§^	0.7 (0.5–1.0)
	Fused group	Preoperative	4.2 ± 1.8	—
		3 days postoperatively	3.6 ± 1.5^†^	3.6 (3.0–4.3)
		3 months postoperatively	1.7 ± 1.0^†,‡^	1.7 (1.4–2.1)
		1 year postoperatively	0.9 ± 0.9^†,‡,§^	1.0 (0.7–1.3)
mJOA scores	Non-fused group	Preoperative	6.1 ± 1.3	—
		3 days postoperatively	7.7 ± 0.9^†^	7.3 (7.1–7.5)
		3 months postoperatively	8.4 ± 0.8^†,‡^	8.1 (7.8–8.3)
		1 year postoperatively	8.8 ± 1.0^†,‡,§^	8.5 (8.1–8.9)
	Fused group	Preoperative	4.7 ± 1.5	—
		3 days postoperatively	6.5 ± 1.1^†^	7.0 (6.8–7.2)
		3 months postoperatively	7.5 ± 0.9^†,‡^	7.8 (7.5–8.1)
		1 year postoperatively	7.9 ± 1.0^†,‡,§^	8.2 (7.8–8.6)
RR (%)	Non-fused group	3 days postoperatively	32.9 ± 8.8	—
		3 months postoperatively	45.4 ± 12.5^‡^	—
		1 year postoperatively	55.3 ± 17.6^‡,§^	—
	Fused group	3 days postoperatively	28.9 ± 6.4	—
		3 months postoperatively	43.0 ± 9.9^‡^	—
		1 year postoperatively	49.7 ± 12.7^‡,§^	—
MacNab criteria	Non-fused group	Excellent (7), good (12), fair (0), poor (0)		
	Fused group	Excellent (5), good (12), fair (1), poor (0)		

VAS, Visual Analog Scale; mJOA, modified Japanese Orthopedic Association scoring system; RR, Recovery Rate. ^†^ Compared to the preoperative value: *p* < 0.05; ^‡^ compared to the postoperative at 3 days value: *p* < 0.05; ^§^ compared to the postoperative at 3 months value: *p* < 0.05.

At the final follow-up, clinical outcomes assessed with the MacNab criteria demonstrated favorable results in both groups: the non-fused group included 7 patients with “excellent” outcomes and 12 with “good” outcomes; the fused group consisted of 5 with “excellent,” 12 with “good,” and 1 with “fair” outcomes(*p* = 0.655).

### 3.3 Radiological outcomes

**Figure 4:**
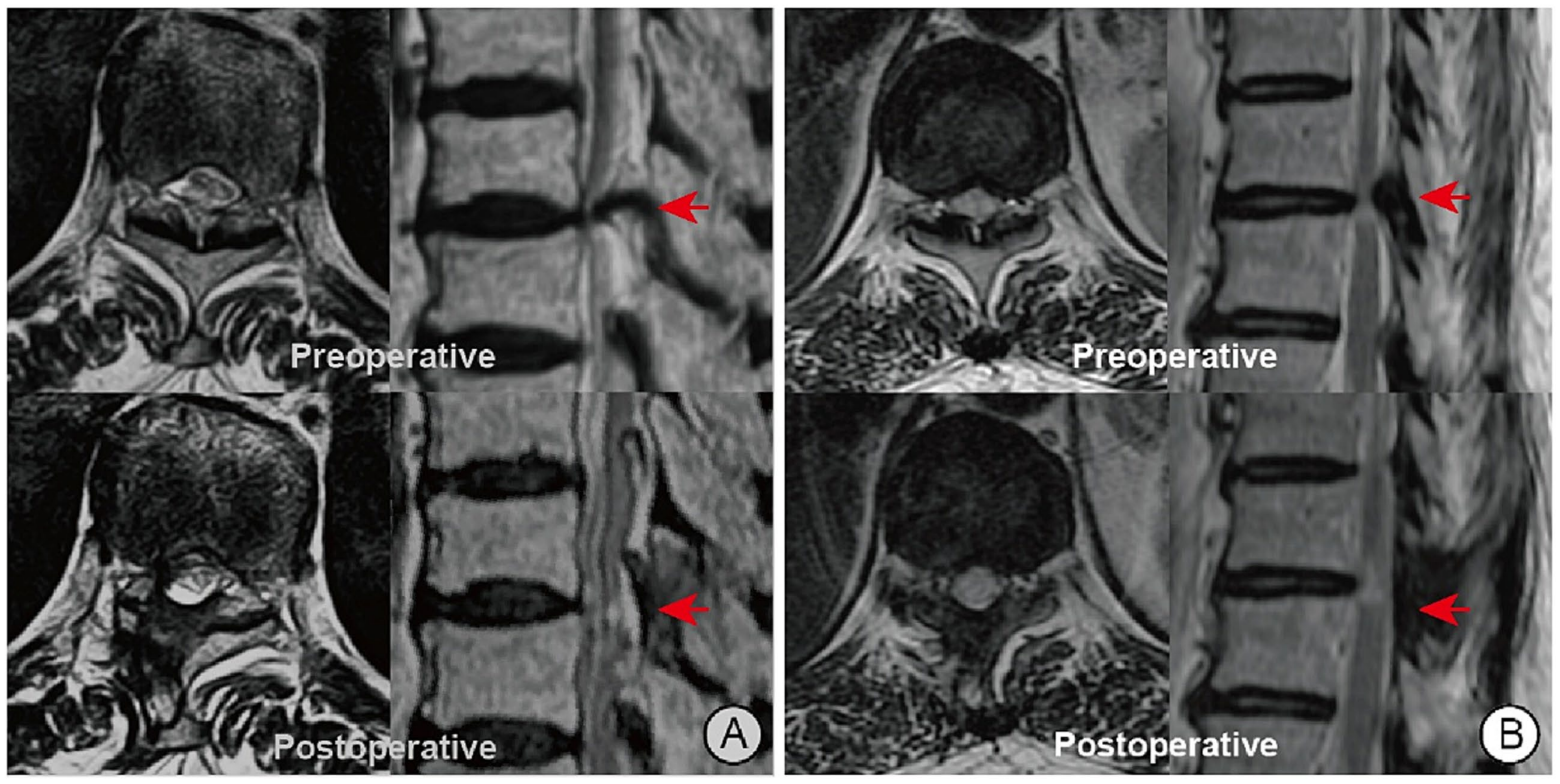
Preoperative and postoperative MRI images of representative cases: fused type **(A)** and non-fused type **(B)**. Spinal cord compression status before and after surgical intervention (red arrows).

Preoperatively, the APD was significantly larger in the non-fused group than in the fused group (*p* < 0.001). Postoperatively, no significant difference in APD was observed between the two groups (*p* = 0.124). Notably, the increase in APD from pre- to postoperative was significantly greater in the fused group than in the non-fused group (*p* < 0.001). No significant correlation was identified between APD increase and clinical outcome improvement (mJOA: *r* = 0.037, *p* = 0.829; VAS: *r* = 0.204, *p* = 0.226). Regarding CSA, the non-fused group had significantly larger CSA than the fused group both preoperatively and postoperatively (*p* < 0.05). However, the increase in CSA was similar between the two groups, with no significant difference (*p* = 0.988). The two observers achieved an ICC > 0.75 (*p* < 0.05), reflecting excellent reliability. Most patients experienced satisfactory decompression, as illustrated by the representative cases (Figure 4 and Table 3).

**Table 3:** Radiographic outcomes.

Variable	Non-fused group (*n* = 19)	Fused group (*n* = 18)	*p* value
APD (mm)			
Preoperative	7.1 ± 1.8	4.4 ± 1.6	<0.001
Postoperative	9.4 ± 1.3	8.7 ± 1.5	0.124
Increase in postoperative	2.3 ± 0.9	4.2 ± 1.2	<0.001
CSA (mm2)			
Preoperative	54.9 ± 19.7	38.6 ± 21.5	0.022
Postoperative	87.7 ± 21.9	71.6 ± 18.8	0.022
Increase in postoperative	32.9 ± 14.1	33.0 ± 17.0	0.988

Data are presented as mean ± standard deviation. APD, anteroposterior diameter; CSA, cross-sectional area.

### 3.4 Complications

**Figure 5:**
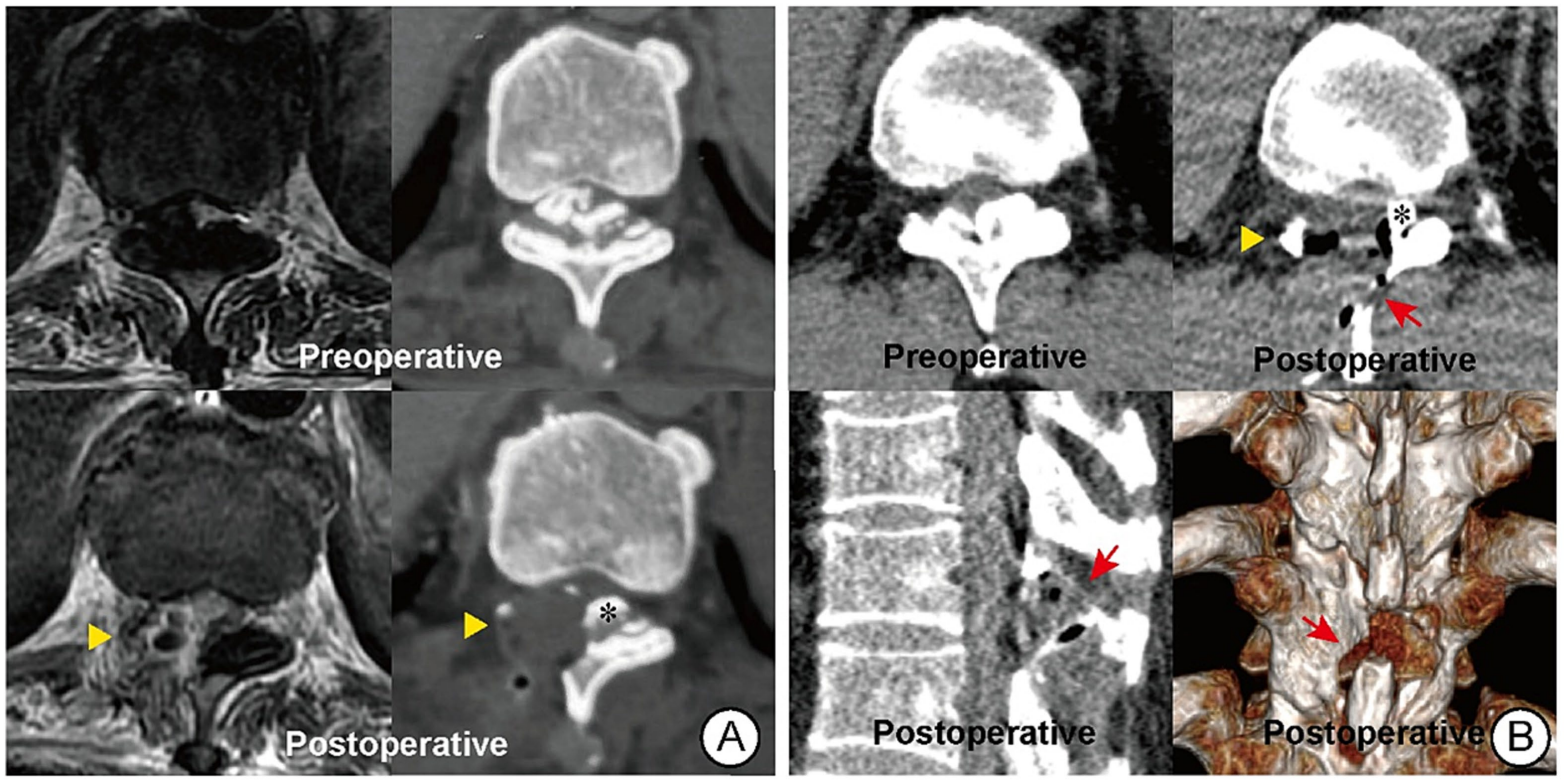
Cases of complications in the fused group. **(A)** Postoperative images showing insufficient contralateral decompression (black asterisk) and excessive ipsilateral facet resection (yellow arrows). **(B)** In addition to inadequate contralateral decompression (black asterisk) and excessive resection of the ipsilateral section (yellow arrows), there is a combination of spinous process fractures (red arrows).

In the fused group, postoperative imaging revealed that two patients had insufficient contralateral decompression and excessive resection of the ipsilateral facet joint. One of these patients had good dural sac expansion postoperatively and significant improvement in myelopathy symptoms. However, a spinous process fracture was also noted. The patient reported slight back discomfort at the final follow-up (Figure 5B). The second patient experienced only mild improvement in symptoms and required walking assistance at the final follow-up (Figure 5A). Neither patient showed signs of postoperative segmental instability. Additionally, six patients in the fused group had intraoperative findings of dural ossification, and among them, two sustained dural tears during decompression. These tears were not repaired intraoperatively because they were small and located at the lateral edge of the dural sac, where attempted suturing could have risked further tearing. No postoperative signs of cerebrospinal fluid leakage were observed; drain output and wound healing remained normal. Postoperative imaging showed no evidence of fluid collection or pseudomeningocele in either patient. During follow-up, neither patient developed any neurological sequelae attributable to the dural tear.

**Figure 6:**
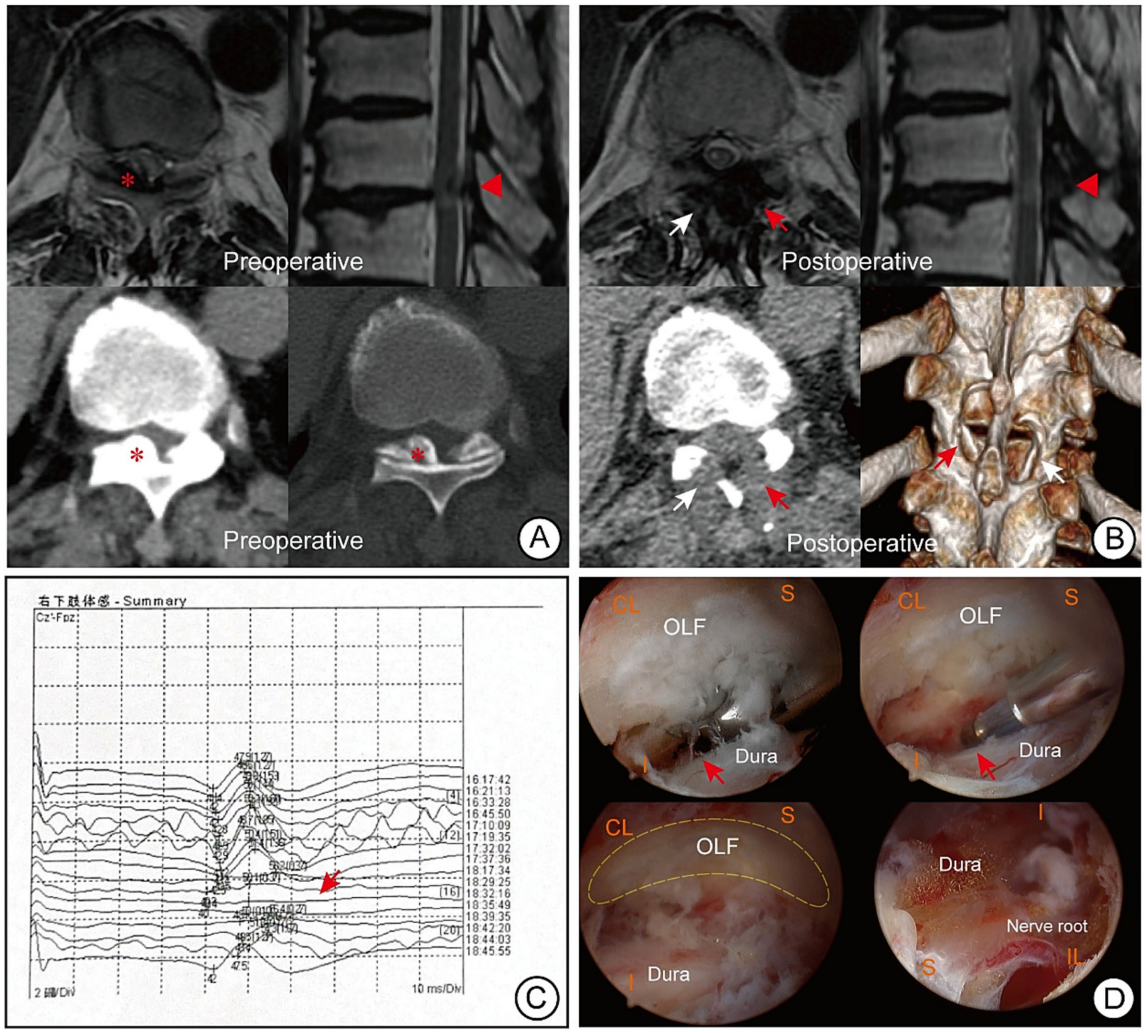
A case of complications in the non-fused group. **(A)** Preoperative images show severe compression on the right side (red asterisk). **(B)** The initial surgery was performed via the left approach (red arrows), followed by postoperative neurological deterioration. A second surgery was then performed using the right approach (white arrows). **(C)** Intraoperative neurophysiological monitoring shows a transient disappearance of the right lower limb somatosensory-evoked potential (red arrows). **(D)** Review of the intraoperative video reveals that the instrument was severely pressed on the dural sac during contralateral decompression (upper) and the residual ossified mass on the contralateral side (lower left). After the second surgery, the contralateral dura and nerve root were completely relieved (lower right). Red arrows: compressed dural sac; yellow dotted line: residual ossified mass.

In the non-fused group, preoperative imaging of one patient showed that the right side of the spinal canal was relatively narrow (Figure 6A). However, muscle strength in the left lower limb (Grade III) was weaker than in the right (Grade IV). Based on this clinical presentation, a left-sided approach was chosen for surgery (Figure 6B). During contralateral decompression, intraoperative neurophysiological monitoring detected a transient disappearance of the right lower limb somatosensory-evoked potential, and neurological deterioration occurred after emerging from anesthesia (Figure 6C). A second decompression was promptly performed using a right-sided approach with biportal endoscopy under local anesthesia. Postoperatively, the patient’s neurological function gradually improved, and by the final follow-up, the patient was able to walk independently without assistance.

All complications were retrospectively classified according to the Clavien-Dindo grading system. Dural tears and stabilized spinous process fractures were classified as Grade I. Cases of insufficient contralateral decompression and facet over-resection, in which no secondary surgery was performed because the patients’ symptoms were tolerable, were provisionally classified as Grade I; however, such cases theoretically carry a long-term risk of progression to Grade IIIb. The case of neurological deterioration requiring a second decompressive procedure was classified as Grade IIIa. No Grade IV or V complications occurred.

## 4 Discussion

Both groups demonstrated significant clinical and radiological improvements from baseline, with similar trajectories of pain relief, functional recovery, and neurological improvement throughout the study period. Notably, previous studies on open laminectomy reported that the recovery rate in the fused group was significantly lower than in the non-fused group (9, 10). Fused OLF is characterized by dense, rigid ossification that often adheres tightly to the dura, typically requiring more extensive decompression (12). Moreover, prolonged spinal cord compression compromises local blood supply and reduces tissue tolerance to surgical trauma, rendering the spinal cord more vulnerable to additional injury (13). Traditional laminectomy for OLF is frequently associated with extensive soft-tissue dissection, longer operative times, substantial blood loss, and an increased risk of spinal cord or nerve root injury, which may raise the incidence of postoperative complications and delay functional recovery (2, 14, 15).

With recent advances in minimally invasive spine surgery, BESS provides a high-definition, magnified surgical field, facilitating a clear distinction between ossified tissue and the normal ligamentum flavum and enabling effective bilateral decompression via unilateral access (8). Continuous saline irrigation through the working channel flushes blood and debris to maintain a clear view. When combined with radiofrequency ablation, this technique achieves precise hemostasis and reduces intraoperative bleeding (16). In addition, the aqueous medium acts as an efficient heat sink, dissipating heat generated during bone resection, thereby protecting neural structures from thermal injury (17). Owing to its minimally invasive nature and effective pain control, BESS may facilitate early return to daily activities and promote faster postoperative recovery (14).

Uniportal endoscopy is another minimally invasive approach for thoracic OLF and has been increasingly accepted in clinical practice (5, 12, 18). Compared with BESS, uniportal techniques offer the advantage of a single, smaller incision and potentially less soft-tissue disruption. However, the single working channel in uniportal endoscopy restricts the visual field and limits instrument maneuverability, which may make contralateral decompression more challenging and prolong operative time (19). By establishing two independent portals, BESS provides a broader operative view and more flexible instrument control, which may help mitigate some of these limitations (8). Nonetheless, both techniques have their own learning curves and technical challenges, and the choice between them may depend on the surgeon’s experience and lesion characteristics.

Dural tears are a major concern in the surgical management of OLF, as they may lead to serious complications such as cerebrospinal fluid leakage, irrigation-related complications, and pseudomeningocele (20). The incidence of dural tears is closely related to the severity of OLF and the presence of dural ossification (17, 21). In this study, two patients with dural ossification sustained dural tears. Owing to the small size and irregular margins of the tears, the limited operative space under endoscopy rendered direct suturing technically challenging and could have increased the risk of neural injury. Therefore, the defects were managed by covering with a gelatin sponge, followed by meticulous layered wound closure. Consistent with recent reports, small dural defects were not directly repaired, and no postoperative cerebrospinal fluid leakage or related complications were observed (16, 21, 22). Nevertheless, larger or irregular dural tears, particularly those associated with neural herniation, warrant a more cautious intraoperative approach. Specifically, tears exceeding 10 mm should be repaired under microscopic guidance, with conversion to open surgery performed when necessary (23).

The use of BESS for severe dural ossification and the tuberous type of OLF remains controversial (24). Some studies suggest that the severely narrowed epidural space and dural adhesions may increase the risk of iatrogenic spinal cord injury and dural tears, thereby recommending open laminectomy as the primary option (8, 16, 25). However, more recent research has provided preliminary evidence supporting the feasibility of BESS, demonstrating that it allows precise dissection between the ossified layer and the dura mater (22, 26). In cases where the ossified tissue is tightly adherent to the dura, a floating technique can be employed to reduce the risk of dural tear (14, 17). During the early clinical phase of our study, due to limited experience with endoscopic management of severe dural ossification, two patients were found to have insufficient contralateral decompression. BESS for severe OLF presents significant technical challenges, particularly for spinal surgeons with limited exposure to endoscopic treatment of ossified lesions (27). To achieve optimal clinical outcomes, surgeons should prioritize adequate decompression and minimizing complications over solely pursuing a minimally invasive approach (21).

In the non-fused group, one patient presented with symptoms that were not fully consistent with the imaging findings. The surgical approach was chosen on the side with more severe symptoms (left) rather than the side with more pronounced cord compression (right). Postoperatively, the patient experienced worsening neurological function in the right lower limb, which was suspected to result from insufficient decompression on the right side. A second decompression was promptly performed using a biportal endoscopic right-sided approach, after which the patient’s neurological function gradually improved. Review of the surgical videos revealed that early exposure of the dura mater limited the working space for instrument manipulation, which was a primary factor contributing to insufficient contralateral decompression. Additionally, repeated contact of surgical instruments with the dural sac during contralateral lamina dissection may have contributed to intraoperative neurophysiological changes and postoperative neurological deterioration (Figure 6D). Even slight mechanical compression during surgery can lead to irreversible spinal cord injury, underscoring the importance of minimizing dural manipulation for the safe removal of OLF (25).

Selecting the appropriate surgical approach is crucial to minimizing the risk of nerve injury, reducing operative time, and optimizing decompression outcomes. The anatomical characteristics of the thoracic vertebrae, coupled with the spinal cord’s limited tolerance to traction, restrict the surgical space and visual field during BESS for thoracic OLF, making contralateral decompression particularly challenging. Our observations suggest that approaching the thinner side of the OLF is technically more demanding than selecting the thicker side, contrary to the findings of a previous study (8). The level of technical difficulty may also be influenced by factors such as the surgeon’s dominant hand, standing posture, and familiarity with left- versus right-sided procedures. Therefore, in cases of asymmetric OLF, a trade-off may exist between the morphologically preferable side and the side that is technically more comfortable for the surgeon. In clinical practice, the optimal approach should be determined by considering both the patient’s anatomical features and the surgeon’s handedness and experience, with surgical safety as the primary priority.

This study had several limitations. First, all procedures were performed by a single surgeon with approximately three years of experience in biportal endoscopic spine surgery, and complications mainly occurred in the first half of the study. This introduces potential learning curve and surgeon-related factors that may have influenced the results, particularly in technically more demanding thoracic cases. Second, in the context of severe myelopathy with a tightly compressed spinal cord, bone work necessitates progressive thinning of the ossified lesion. However, under continuous endoscopic irrigation, bone dust is rapidly washed away, making it difficult to visually confirm the ongoing drilling. Consequently, the surgeon must rely heavily on tactile feedback during this critical step. This highlights an inherent limitation of the endoscopic environment and emphasizes the importance of surgeon experience, as well as the use of instruments that provide reliable tactile feedback to ensure safe and controlled decompression. Finally, this was a retrospective, single-center study, which may have introduced selection bias. The sample size was relatively small and the follow-up period limited, which precluded evaluation of long-term outcomes such as recurrence, kyphosis, or segmental instability. Future studies with extended follow-up and larger prospective controlled trials are warranted to address these issues and validate our findings.

## 5 Conclusion

BESS yields favorable short-term clinical outcomes in both fused and non-fused thoracic OLF. Notably, the fused group had longer operative time and a higher complication rate. For complex cases—particularly fused-type OLF with severe spinal cord compression—thorough preoperative evaluation is advisable. Given the technical demands, these endoscopic procedures may be best performed by surgeons with adequate experience.

## Data Availability

The original contributions presented in the study are included in the article/supplementary material, further inquiries can be directed to the corresponding authors.
